# How Metacognitions Contribute to Compulsive Online Shopping: An Exploratory Study

**DOI:** 10.1002/jclp.23752

**Published:** 2024-11-11

**Authors:** Giulia Fioravanti, Marcantonio M. Spada, Sara Bocci Benucci, Silvia Casale, Alessio Gori

**Affiliations:** ^1^ Department of Health Science University of Florence Firenze Italy; ^2^ Division of Psychology, School of Applied Sciences London South Bank University London; ^3^ Department of Experimental and Clinical Medicine University of Florence Florence Italy

**Keywords:** boredom proneness, compulsive online shopping, impulsivity, materialism, metacognitions

## Abstract

**Objectives:**

Compulsive Online Shopping (COS) is considered a technological addiction, characterized by excessive engagement in online shopping behaviors that can cause economic, social, and emotional impairments in an individual's life. Among the theoretical models aimed at conceptualizing addictive behaviors, the metacognitive model has gained increased attention. However, no previous study has investigated the role of metacognitions in COS. The current study was aimed at clarifying the contribution of metacognitions about online shopping as potential mediating variables in the relationship between some well‐established psychological correlates (i.e., boredom proneness, impulsivity, materialism, negative affect) and COS.

**Methods:**

A sample of 254 participants (mean age = 34.79 ± 11.45; Females = 84.3%) was recruited using convenience sampling.

**Results:**

The hypothesized model produced a good fit to the data and accounted for 48% of COS variance. All the correlates (i.e., boredom proneness, impulsivity, materialism, and negative affect) were significantly and positively associated with Positive Metacognitions About Emotional And Cognitive Regulation, which in turn predicted COS. Boredom proneness and impulsivity were also positively associated with Negative Metacognitions About Uncontrollability And Cognitive Harm of online shopping, which in turn predicted COS. All the indirect effects were significant.

**Conclusions:**

The present findings add to the argument that the metacognitive model of addictive behaviors may applied to the understanding of COS and open the possibility of applying metacognitive techniques to the treatment of COS.

## 1 Introduction

Shopping is an activity driven by utilitarian but also recreational and leisure motivations (Rose and Dhandayudham 2014). However, from the beginning of the 20th century, scientific research (e.g., Edwards 1993; Müller, Mitchell, and de Zwaan 2015; Niedermoser et al. 2021) has identified a “dark side” to shopping, namely compulsive shopping, a problematic condition characterized by loss of control over the shopping behavior, craving for shopping and excessive concern about shopping when not making purchases (Andreassen et al. 2015; Müller, Mitchell, Zwaan et al. 2015). Moreover, in compulsive shopping, shopping continues despite its negative consequences (e.g., financial problems, relational conflicts, and psychological distress) (see Niedermoser et al. 2021; Aboujaoude 2014 for reviews). Although compulsive shopping is not included in DSM5 “Substance‐Related and Addictive Disorders” chapter (American Psychiatric Association 2013), some authors have suggested that compulsive buying‐shopping disorder may fit with the ICD‐11 category “other specified disorders due to addictive behaviors” (Brand et al. 2020). This suggestion is based on empirical evidence highlighting that: (i) this behavioral pattern leads to clinically significant distress and impairments in everyday life; (ii) this behavior can be appropriately explained by addiction framework theories; and (iii) some of the mechanisms involved share similarities to those in other addictive behaviors (e.g., Brand et al. 2019; Müller et al. 2019; Raab et al. 2011). In particular, evidence from community and clinical samples shows that COS shares some key features of gambling and gaming disorder, including diminished control, increasing priority to the extent that the activity interferes with other interests or daily responsibilities (see Black 2022), impairment in personal, family, social, educational, occupational, or other important areas of functioning (e.g., Achtziger et al. 2015). Moreover, similar to addictive behaviors, online buying‐shopping activities are continued or even escalated despite negative consequences (e.g., indebtedness, clinically significant distress; Müller et al. 2021).

Nowadays, with the spread of the Internet and the growth of e‐commerce activities, individuals can shop at any time without leaving their homes. Additionally, the advent of the COVID‐19 pandemic and the related measures implemented by governments to address the spread of the virus, including the temporary closing of shops, may have accelerated the shift to online shopping, contributing to a change in individuals' shopping habits (see for instance, Georgiadou et al. 2021; Maraz, Katzinger, and Yi 2021). All these aspects have prompted researchers to consider problematic conditions related to online shopping and, in particular, Compulsive Online Shopping (COS).

COS is considered a technological addiction (e.g., Rose and Dhandayudham 2014) characterized by excessive engagement in online shopping behaviors that can cause economic, social, and emotional impairments in an individual's life (Andreassen et al. 2015). Diminished control over buying behavior, salience, tolerance, craving responses, and motivation and compensation processes have been found among people with COS (e.g., Brand et al. 2020; Müller et al. 2019; Starcke et al. 2018). Negative outcomes, including legal and financial difficulties, alterations in sleep patterns, somatic symptoms, lower life satisfaction, and impaired quality of life, have been observed (e.g., Muller, Joshi, and Thomas 2022). Although they share the “object” of the addiction, compulsive buying and COS are different in many respects, with the Internet environment offering features that may increase the chances of compulsive behaviors. First, while traditional shopping is a social activity involving personal interaction with others, a key feature of online shopping is social anonymity, which may encourage more excessive behavior through the (well‐established) online disinhibition effect (see Sun and Wu, 2011). Second, the Internet environment allows one to approach a wider and more varied spectrum of products to shop faster at any time and place. Related to this point, the frequent and constantly changing stimuli provide repeated cognitive stimulations that may create cognitive overload, which, in turn, makes temptation harder to resist (Fudenberg & Levine, 2006).

A limited number of studies have addressed the prevalence of COS in representative samples, reporting a prevalence rate of about 3−4% (e.g., Adamczyk 2021; Augsburger et al. 2020). Moreover, inconsistent evidence about the role of gender and age exists. Some studies found that women are more vulnerable to COS (e.g., Adamczyk 2021), whereas others did not (e.g., Augsburger et al. 2020). Age has not always been found to be negatively correlated with COS (Augsburger et al. 2020), contradicting the results of a meta‐analysis in which being young and female were associated with an increased tendency toward COS (Maraz, Griffiths, and Demetrovics 2016).

Based on empirical findings (e.g., Rose and Dhandayudham 2014), recognized predictors for COS appear to comprise low self‐esteem, low self‐regulation, negative emotional state, boredom proneness, and impulsivity (i.e., recognized predictors of addictive behaviors in general) as well as some specific predictors like strong materialistic values tendency (e.g., Dittmar, Long, and Bond 2007). As already said specific motives which appear to prompt COS include the possibility of buying without being observed by others, avoiding social interactions while shopping, experiencing speedy positive feelings, and the possibility of having access to a wide range of products (Kukar‐Kinney, Ridgway, and Monroe 2009). Alongside the motive of seeking gratification and pleasure from online shopping, the expectancy of relief from negative feelings and escape from unpleasant emotions when using online shopping sites (compensation) may prompt COS (e.g., Trotzke et al. 2015). Furthermore, identity‐seeking motives (i.e., the need to enhance one's identity through material goods) could be associated with COS, especially among individuals with strong materialistic values (e.g., Dittmar, Long, and Bond 2007).

### 1.1 The Metacognitive Model of Addictive Behaviors

Among the theoretical models aimed at explaining addictive behaviors, the metacognitive model of addictive behaviors (Spada et al. 2015) has gained rising attention and empirical evidence for both substance‐related and behavioral addictions (e.g., Hamonniere and Varescon 2018; Casale, Musicò, and Spada 2021). Metacognition can be defined as beliefs and cognitive processes involved in the appraisal, control, and monitoring of thinking (Flavell 1979; Wells 1995). In the context of addictive behaviors, two types of metacognitions were found to be particularly relevant: (i) generic metacognitions about cognitive‐affective experiences (e.g., beliefs about the need to control thoughts); and (ii) specific metacognitions related to the addictive behavior (i.e., beliefs about the effect of engaging in addictive behavior on emotion and cognition). The latter are divided into (a) *positive metacognitions*, referring to beliefs about the usefulness of the addictive behavior in regulating unpleasant cognitive and emotional experiences (e.g., “Alcohol helps me to reduce my anxiety”); and (b) *negative metacognitions*, concerning beliefs about the negative effect of the addictive behavior on cognitive functioning/control (e.g., “Alcohol use will damage my memory capacities”), and about the uncontrollability of thoughts related to the addictive behavior and over the behavior itself (e.g., “Thoughts about using alcohol control my mind”) (for a review see Hamonniere and Varescon 2018). According to the triphasic metacognitive model of addictive behaviors (Spada, Caselli, and Wells 2013), positive metacognitions play a central role in the initiation of addictive behavior (i.e., the pre‐engagement phase) since they motivate individuals to engage in it as a strategy to cope with negative thoughts and emotions. Negative metacognitions are activated in the engagement and post‐engagement phases of the addictive behavior and play a role in its maintenance. Specifically, the perception of a lack of control over addiction‐related thoughts and behaviors elicits negative emotional states that individuals try to alleviate by continuing to engage in the addictive behavior (Spada, Caselli, and Wells 2013). As previously stated, empirical evidence about the role of generic and addictive behavior‐related metacognitions emerged in both substance‐based addictive behaviors and behavioral addictions (Hamonniere and Varescon 2018). Furthermore, a very recent systematic review confirmed a positive association between metacognitions and four technological addictions (Internet Gaming Disorder, problematic Internet use, problematic Smartphone use, and problematic social networking sites use).

### 1.2 The Current Study

**Figure 1:**
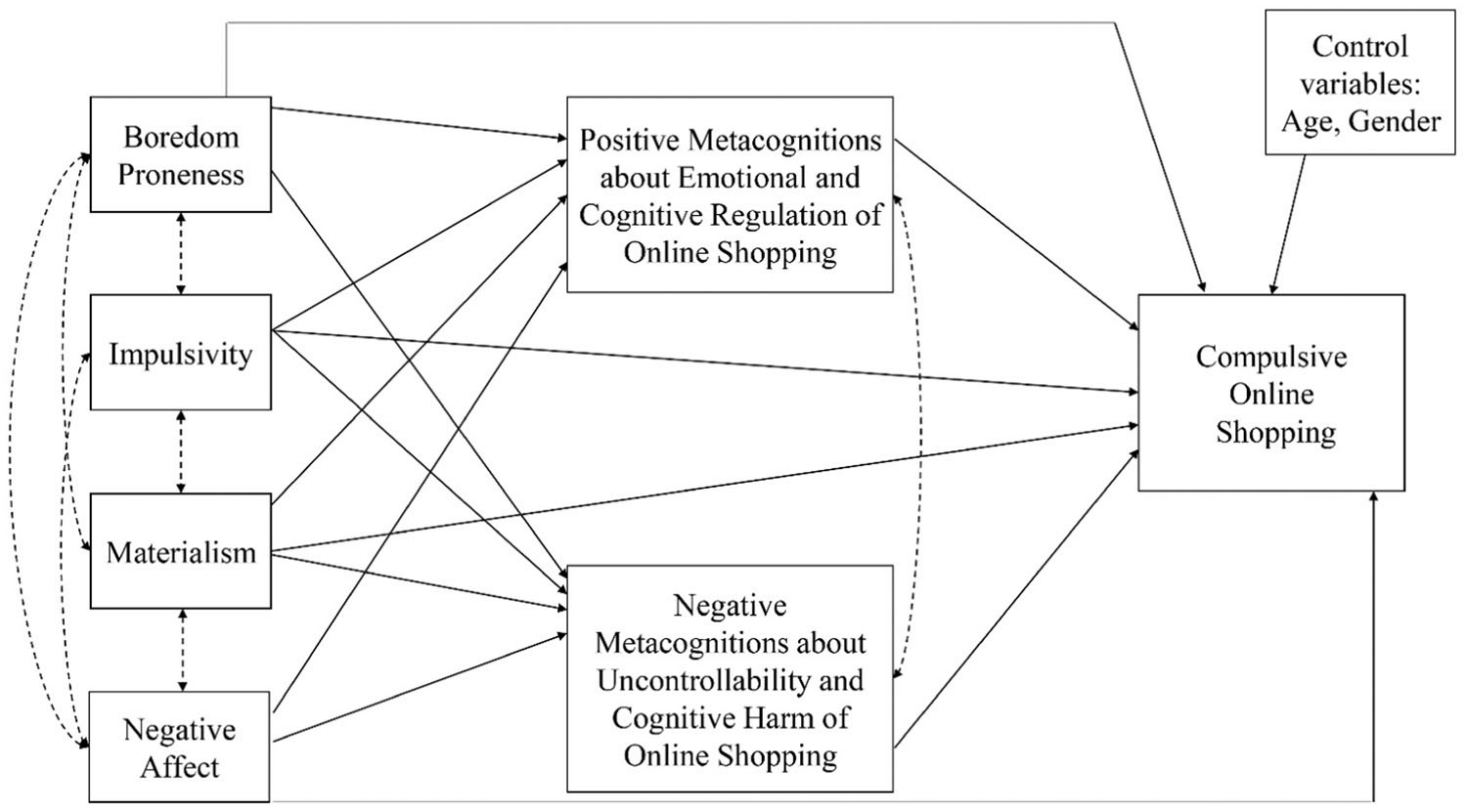
Proposed theoretical model.

Metacognitions seem to play a mediating role in the association between psychological risk factors (e.g., anxiety and depressive symptoms) and addictive behaviors (e.g., Spada and Wells 2005; Spada et al. 2007). In the case of technological addictions, individuals with high levels of negative emotional states seem at higher risk of developing an unregulated use of the Internet, online gaming, social networking, or other online activities as they believe that engaging in these behaviors can help them to find relief from these negative internal states (i.e., positive metacognitions about the addictive behavior). Furthermore, beliefs about the lack of control over the problematic behavior (i.e., negative metacognitions about the addictive behavior) reinforce the addictive behavior itself (Casale, Fioravanti, and Spada 2021). The link between negative emotional states and COS is well‐established, whereas no previous study has investigated the role of metacognitions as potential mediating variables in the link between these well‐known correlates and compulsive buying in the online environment. The current study aims to fill the identified gap in the literature by evaluating the applicability of the metacognitive model of addictive behavior to COS. In particular, we aimed to investigate the role of positive and negative metacognitions as potential explanatory mediating variables in the association between some well‐established psychological correlates (i.e., boredom proneness, impulsivity, materialism, negative affect) and COS. The proposed model is displayed in Figure 1. Specifically, we hypothesized that all the predictive variables (i.e., boredom proneness, impulsivity, materialism, and negative affect) would predict COS both directly and indirectly through (i) positive metacognitions about the usefulness of online shopping to regulate emotional and cognitive states; and (ii) negative metacognitions about the uncontrollability and cognitive harm of online shopping. Given the previous contradictory results, age and gender would be treated as control variables in the model.

## 2 Methods

### 2.1 Participants and Procedure

An a priori analysis was conducted using G*Power (Faul et al. 2009) to determine sample size adequacy. The results indicated that 141 participants would be necessary to achieve a power of 0.95 by assuming a medium effect size (*f* ^2^ = 0.20) and an *α* level of 0.05. A sample of 254 participants (mean age = 34.79 ± 11.45; age range = 19−75, Females = 84.3%) was recruited through announcements on (i) Facebook thematic groups, (ii) Instagram stories/Direct messages, (iii) Telegram and WhatsApp messages, using a convenience sampling approach; only participants who declared to engage in online shopping were included in the study. The sample was mainly composed of employees (42.6%), unemployed (20.9%), students (18.9%), entrepreneurs/freelancers (15.7%), and retired (2.0%) individuals. Concerning educational background, 48.8% of the sample reported having a high school diploma, 21.30% a master's degree, 19.7% a bachelor's degree, 5.5% a middle school diploma, 3.6% a higher qualification (e.g., PhD), and the remaining 1.2% declared “other.” Considering participants' yearly household income, 55.1% claim to earn 10.000€−30.000€, 21.7% 30.000€−50.000€, 13.8% 0€−10.000€, 5.8% 50.000€−70.000€, 2.1% 70.000€−100.000€ and 0.5% over 100.000€, a year.

Participants were informed that participation was voluntary and anonymous and that confidentiality was guaranteed. A web link directed the participants to the study website, and if they consented to participate, they were asked to answer demographic questions and a batch of self‐report questionnaires. No remunerative rewards were given. The study procedures were carried out in accordance with the Declaration of Helsinki. The Institutional Review Board of the University of [first author] approved the study.

### 2.2 Measures

#### 2.2.1 Online Shopping Habits

Participants were asked to respond to some questions investigating their online shopping habits. Specifically, they were asked to indicate (i) how long they have been doing online shopping; (ii) how much time they spend daily doing online shopping, (iii) how often they do online shopping, (iv) how much on average they usually spend in a month, (v) which type of products they usually buy, and (vi) if the frequency of their online shopping has increased since the beginning of the COVID‐19.

#### 2.2.2 Boredom Proneness

Boredom proneness was assessed using the 8‐item Boredom Proneness Scale–Short Form (BPS‐SF; Struk et al. 2017), which is a short version of the original BPS (Farmer and Sundberg 1986). The BPS‐SF uses a 7‐point Likert scale ranging from 1 (*Highly disagree*) to 7 (*Highly agree*); a sample item is: “I find it hard to entertain myself.” The total score is obtained by summing the response to all items included in the scale, and higher scores indicate higher boredom proneness. The translation of the items for the short form was obtained using the BPS–Italian version (Craparo et al. 2013), which demonstrated good psychometric properties. In the current sample, Cronbach's *α* for the BPS‐SF was 0.92.

#### 2.2.3 Impulsivity

Impulsivity was measured using the Italian version (Spinella 2007) of the 15‐item Barratt Impulsivity Scale (BIS‐15). The BIS‐15 uses a 4‐point Likert scale ranging from 1 (*Rarely/never*) to 4 (*Almost always*), and a sample item is: “I buy things on impulse.” The scale maintains the 3‐factor structure (non‐planning, motor impulsivity, and attention impulsivity) of the original 30‐item version (Patton, Stanford, and Barratt 1995). The scale is composed of six reversed items and the total score can be computed by summing the response to all the items. Higher scores indicate a higher level of impulsivity. In the current sample, Cronbach's *α* for the BIS‐15 was 0.80.

#### 2.2.4 Materialism

Materialism was assessed using the 18‐item Material Values Scale (MVS; Richins and Dawson 1992). The scale was translated from English into Italian according to the recommendations of the International Test Commission (2005) since a validated Italian version is not available. The Italian translation was obtained from the parent English version using a standard back‐translation technique (Brislin 1986). A bilingual individual who was unaffiliated with the study translated the scale from English to Italian; a second individual then translated the version back to English. Minor discrepancies were settled through consensus. Participants are asked to rate the items on a 5‐point Likert scale ranging from 1 (*Totally disagree*) to 5 (*Totally agree*), and a sample item is: “The things I own say a lot about how well I'm doing in life.” The scale comprises three factors: success, centrality, and happiness. There are seven reversed items and the total score can be computed by the mean of the response to all the items. Higher scores indicate higher materialism. In the current sample, Cronbach's *α* for the MVS was 0.76.

#### 2.2.5 Negative Affect

Negative affect was assessed using the Italian version (Terracciano, McCrae, and Costa 2003) of the subscale *Negative Affect* of the Positive and Negative Affect Schedule (PANAS; Watson, Clark, and Tellegen 1988). The subscale is composed of 10‐item which are presented by asking the responders how much it feels represented by an adjective on a 5‐point Likert scale ranging from 1 (*at all*) to 5 (*a lot*). Samples of adjectives are “hostile” and “nervous.” The total score is obtained by computing the responses to all the items, and higher scores indicate higher negative affect. In the current sample, Cronbach's *α* for the subscale negative affect of the PANAS was 0.89.

#### 2.2.6 Metacognitions about Online Shopping

Metacognitions about online shopping were measured using a modified version of the Italian Metacognitions about Smartphone Use Questionnaire (MSUQ; Casale, Caponi, and Fioravanti 2020). Specifically, 21 items were used and adapted to be applied to online shopping instead of Smartphone use. Participants are asked to respond on a 4‐point Likert scale ranging from 1 (*Do not agree*) to 4 (*Agree very much*). The questionnaire is composed of two subscales named *Positive Metacognitions about Emotional and Cognitive Regulation* (a sample item is: “Doing online shopping reduces my anxious feelings”), and *Negative Metacognitions about Uncontrollability and Cognitive Harm* (a sample item is: “My thoughts about doing online shopping are becoming an obsession). In the current sample, Cronbach's *α* for the two subscales were 0.95 (PM ECR) and 0.94 (NM UH).

#### 2.2.7 COS

COS was assessed using the Italian version (Gori, Topino, and Casale 2022) of the 28‐item COS Scale (COSS; Manchiraju, Sadachar, and Ridgway 2017). The COSS is a theoretically guided, culturally neutral self‐report scale to assess the levels of COS. It was developed by adapting the initial pool of 28 items of the Bergen Shopping Addiction Scale (Andreassen et al. 2015) to reflect COS—that is, the content was slightly modified to obtain an exclusive focus on online behavior. The COSS meets the addiction criteria (e.g., salience, mood modification, etc.) established in the DSM‐5 (American Psychiatric Association 2013). Participants are asked to respond on a 7‐point Likert scale ranging from 1(*Highly disagree*) to 7 (*Highly agree*). When computing the score, items can be grouped into seven subdimensions, in line with the addiction criteria established in the DSM‐5 (American Psychiatric Association 2013), reflecting the components of Salience (e.g., “Online shopping/buying is the most important thing in my life.”), Mood modification (e.g., “I shop/buy things online to forget about personal problems.”), Conflict (e.g., “I give less priority to hobbies, leisure activities, job/studies, or exercise because of online shopping/buying”), Tolerance (e.g., “I spend more and more time shopping/buying online.”), Relapse (e.g., “I have tried to cut down on online shopping/buying without success.”), Withdrawal (e.g., “I become stressed if obstructed from shopping/buying things online”), and Problems (e.g., “I shop/buy online so much that it has caused economic problems”). Additionally, the total score is obtained by computing the responses to all the items, and higher scores indicate higher COS. In the current sample, Cronbach's *α* for the total COSS was 0.97.

The survey was tested on a small convenience sample of 15 adults to identify problems before implementing the study.

### 2.3 Data Analysis

Descriptive statistics and Pearson's Product‐Moment correlations between the study variables were computed. The pattern of relationships specified by our hypothesized model (Figure 1) was tested using a path analysis through the lavaan package (Rosseel 2012) of R software with the Robust Maximum Likelihood (RML) estimation method. In our model, boredom proneness, impulsivity, materialism, and negative affect were the predictive variables; Positive Metacognitions about Emotional and Cognitive Regulation and Negative Metacognitions about Uncontrollability and Cognitive Harm were the mediators; and COS was the outcome variable with age and gender as control variables. Indirect effects were evaluated with the distribution of the product of coefficients (MacKinnon et al. 2002; MacKinnon Lockwood, and Williams 2004). We first tested the full model and then removed step‐by‐step path coefficients not significant at the 5% level to select the most plausible model. To evaluate the model's goodness of fit, we considered the *χ* ^2^ (and its degrees of freedom and *p* value), the Standardized Root Mean Square Residual (SRMR—Jöreskog and Söbom 1993) “close to” 0.09 or lower, the Comparative Fit Index (CFI—Bentler 1995) “close to” 0.95 or higher (Hu and Bentler 1999), and the Root Mean Square Error of Approximation (RMSEA—Steiger 1990) less than 0.08 (Browne and Cudeck 1993).

## 3 Results

### 3.1 Descriptive and Correlational Analyses

Participants' online shopping habits are shown in Table 1, whereas descriptive statistics and Pearson's Product‐Moment correlations among the study variables are presented in Table 2.

**Table 1:** Participants' online shopping habits.

	% (*n* = 254)
*How long have you been doing online shopping?*	
Less than 6 months	5.9%
6 months−1 year	4.7%
1−2 years	17.3%
2−5 years	33.1%
more than 5 years	39.0%
*How much time do you spend daily doing online shopping?*	
Less than half an hour	76.8%
half an hour−1 h	18.1%
1−2 h	3.5%
2−3 h	1.2%
3−4 h	0.4%
More than 4 h	/
*How often do you do online shopping?*	
Less than 1 a month	31.5%
About once a month	34.6%
About once every 2 weeks	18.5%
About once a week	11.4%
Every 2−3 days	3.5%
Everyday	0.4%
*How much do you usually spend on average in a month for online shopping?*	
Less than 50€	35%
50−100€	42.9%
100−300€	18.5%
300−500€	3.1%
500−1.000€	0.4%
over 1.000€	/
*Which type of products do you usually buy? (possibility of select more than one option)*	
Clothes	70.7%
Household items	44.0%
Eletronic devices/appliances	39.8%
Books/CDs/Newspapers/Magazines	38.2%
Travels (accomodation)	22.8%
Tickets (means of transport, concerts)	21.3%
Food	13.4%
Softwares	5.5%
Other	11%
*Since the COVID‐19 pandemic began, has the frequency with which you shop online increased?*	
Yes	70.50%
No	29.50%

**Table 2:** Means, standard deviations, range, skewness, kurtosis and Pearson's product‐moment correlations among study variables.

	*M*	SD	Range	Skewness	Kurtosis	1	2	3	4	5	6	7	8	9
1. Age	34.79	11.45	19−75			—								
2. Gender	—	—	—			−0.01	—							
3. Boredom proneness	22.74	11.71	7−56	0.70	−0.40	−0.34**	0.06	—						
4. Impulsivity	30.57	6.17	15−60	0.57	0.41	0.00	0.13*	0.39**	—					
5. Materialism	58.72	9.53	18−90	−0.10	0.09	−0.24**	−0.03	0.17**	0.18**	—				
6. Negative affect	22.08	8.16	10−50	0.49	−0.54	−0.18**	0.11	0.46**	0.39**	0.09	—			
7. Positive Metacognitions about Emotional and Cognitive Regulation	15.46	6.67	11−44	2.09	4.60	−0.14*	0.12	0.38**	0.39**	0.28**	0.35**	—		
8. Negative Metacognitions about Uncontrollability and Cognitive Harm	11.60	4.00	10−40	3.42	12.98	−0.04	0.04	0.30**	0.34**	0.14*	0.30**	0.52**	—	
9. Compulsive Online Shopping	46.47	26.60	28−196	2.64	8.73	−0.16**	0.13*	0.42**	0.38**	0.23**	0.33**	0.59**	0.59**	—

* *p* < 0.05;

** *p* < 0.001.

Boredom proneness, impulsivity, materialism, and negative affect were positively associated with positive and negative metacognitions and COS. Both positive and negative metacognitions about online shopping were positively associated with COS. Age was negatively correlated with COS, whereas gender (female) was positively correlated.

### 3.2 Path Analysis

**Figure 2:**
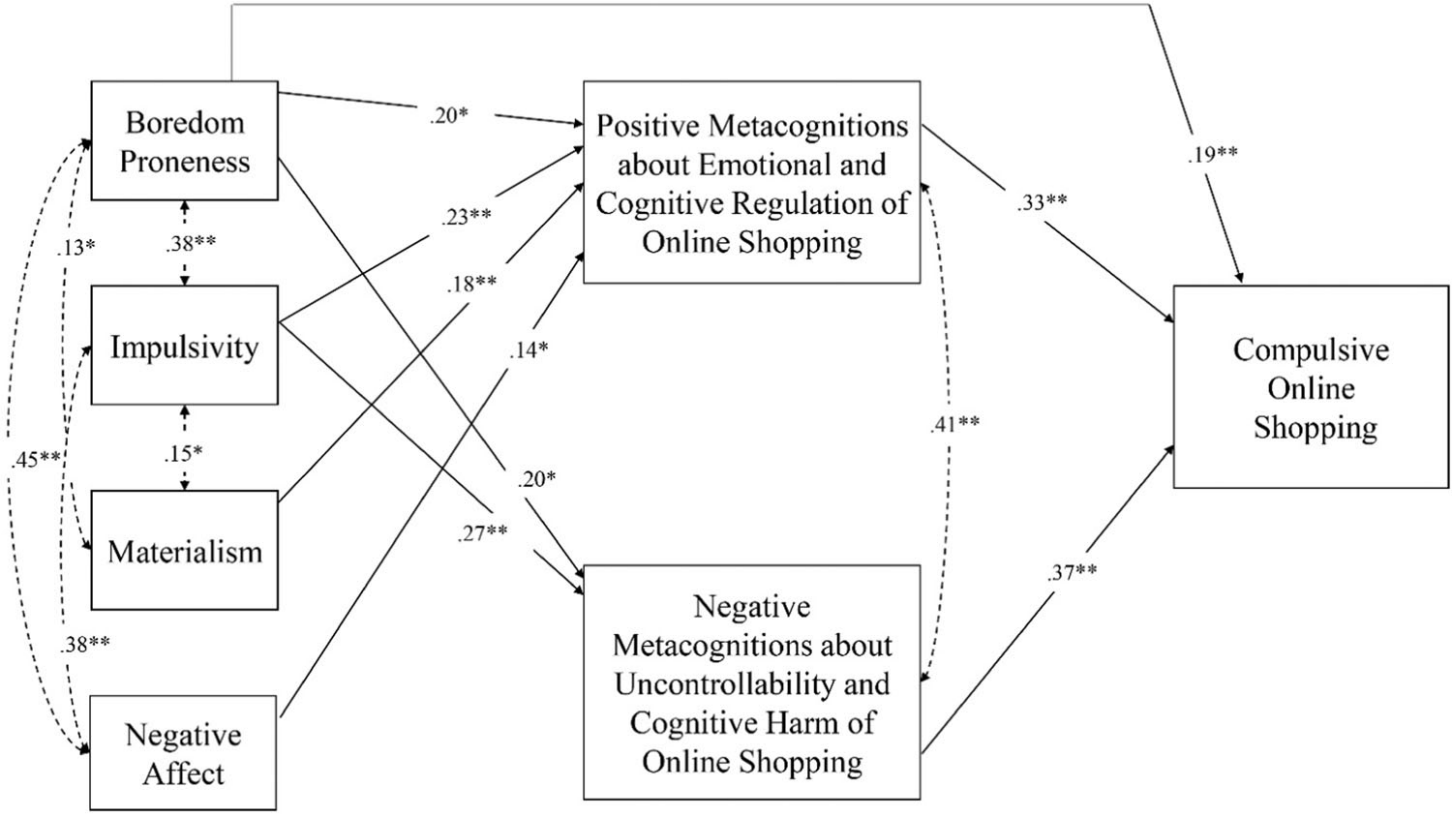
Results of the path analysis.

The tested model accounted for 48% of the variance in COS and showed good fit indices: *χ* ^2^ = 8.013, *df* = 6, *p* = 0.02; RMSEA [90% CI] = 0.04 [0.00−0.09]; CFI = 0.99; SRMR = 0.04 (Figure 2). Boredom proneness, impulsivity, materialism, and negative affect predicted Positive Metacognitions about Emotional and Cognitive Regulation, which, in turn, positively predicted COS. Boredom proneness and impulsivity also predicted Negative Metacognitions about Uncontrollability and Cognitive Harm, which, in turn, predicted COS. All the indirect effects were significant (see Table 3). Additionally, a significant direct effect of boredom proneness on COS was found.

**Table 3:** Indirect effects of the path analysis.

	Estimate	SE	95% CI	Standardized	Product of coefficients (*P*)
Boredom Proneness → Positive Metacognitions about Emotional and Cognitive Regulation → Compulsive Online Shopping	0.145	0.052	0.04−0.24	0.06	19.08*
Impulsivity → Positive Metacognitions about Emotional and Cognitive Regulation → Compulsive Online Shopping	0.325	0.101	0.12−0.52	0.07	22.96*
Materialism → Positive Metacognitions about Emotional and Cognitive Regulation → Compulsive Online Shopping	2.852	0.953	0.98−4.71	0.06	20.76*
Negative Affect → Positive Metacognitions about Emotional and Cognitive Regulation → Compulsive Online Shopping	0.150	0.066	0.02−0.28	0.05	14.59*
Boredom Proneness→ Negative Metacognitions about Uncontrollability and Cognitive Harm → Compulsive Online Shopping	0.163	0.057	0.05−0.27	0.07	22.10*
Impulsivity → Negative Metacognitions about Uncontrollability and Cognitive Harm → Compulsive Online Shopping	0.419	0.116	0.19−0.64	0.09	29.23*

* *p* < 0.05.

## 4 Discussion

The present study aimed to explore the role of positive and negative metacognitions as potential mediators in the association between some well‐established individual differences (i.e., boredom proneness, impulsivity, materialism, and negative affect) and COS. In accordance with previous studies (e.g., Çelik and Köse 2021; Georgiadou et al. 2021; Maraz, Katzinger, and Yi 2021), two‐thirds of the sample reported an increase in time spent on online shopping after the onset of the COVID‐19. The restrictions due to the COVID‐19 pandemic and widespread e‐commerce habits may have changed consumers' behaviors by enhancing the transition from in‐store to online shopping. The easy access to shopping websites, mobile payment technologies, and the possibility of browsing online stores at any time and in any situation (e.g., while at work, at school, or on public transport) may render some vulnerable individuals at higher risk of developing COS.

With regard to the psychological factors that might be positively associated with COS, the findings of the current study confirm the role of impulsivity, boredom proneness, materialism, and negative affect in contributing to the phenomenon (e.g., Muller, Joshi, and Thomas 2022). Additionally, the effect of all these variables was mediated by metacognitions about online shopping. This result extends previous findings by evidencing a potential explanatory mechanism (i.e., metacognitions) that has already been found to be involved in various addictive behaviors but has never been studied in COS. Specifically, we found that (i) Positive Metacognitions about Emotional and Cognitive Regulation in online shopping play a mediating role in the association between boredom proneness, impulsivity, materialism, negative affect, and COS, and (ii) Negative Metacognitions about Uncontrollability and Cognitive Harm of online shopping only play a mediating role in the association between boredom proneness, impulsivity, and COS.

The perception of internal negative states (i.e., boredom, negative affect) could lead to COS by activating beliefs that online shopping is useful in regulating negative emotions and it is an effective way to cope with these states. Expectancies of relief from undesirable feelings and escape from negative affect have already been linked to COS (e.g., Trotzke et al. 2015). However, positive metacognitions differ from expectancies in that they are explicitly focused on how engagement in the behavior can be useful to achieve cognitive and emotional self‐regulation (i.e., thinking about the behavior as a coping strategy to manage negative thoughts and emotions) (e.g., Spada et al. 2015). On the other hand, positive expectancies refer to the general anticipation of the positive consequences of the behaviors (e.g. “I shop online to experience pleasure”). Furthermore, the mediating role of positive metacognitions in the relationship between impulsivity and COS could be explained by the fact that impulsive individuals may resort to maladaptive coping mechanisms, such as COS, as they think that online shopping might be effective in alleviate distress in the short term. The role of positive metacognitions in contributing to addictive behaviors has been previously reported in the context of different behavioral addictions (e.g., Spada et al. 2015; Casale, Rugai, and Fioravanti 2018; Casale, Fioravanti, and Spada 2021). The current findings extend their role in COS suggesting the applicability of the metacognitive model for the understanding of this phenomenon.

The finding about the role of Positive Metacognitions about Emotional and Cognitive Regulation (e.g, online shopping makes my worries more bearable, online shopping distracts my mind from problems) in explaining the association between materialism and COS is of particular interest since it offers a possible explanation for the effect of an addictive behavior‐specific psychological correlate. The greater the importance attached to material objects, the greater the belief that buying goods will help to regulate emotions and cognitions. These might be related to reaching happiness and life satisfaction and improving self‐esteem by acquiring material objects. Beliefs that online shopping reduces anxious feelings and worries may be activated to reduce these negative affective states fueled by the perceived self‐discrepancy between actual and ideal identity (i.e., enhanced by material goods), leading, over time, to a loss of control over online shopping behavior. This is consistent with the observation that identity‐seeking motives could lead to COS in people with high levels of materialism (e.g., Dittmar, Long, and Bond 2007). It has been suggested that specific objects of addiction do not play a central role in the development of addiction, and evidence supports a syndromal view of addiction (Shaffer et al. 2004). Our study seems to suggest that specific pathways to specific expressions of addiction could be identified by taking into account premorbid individual differences, in keeping with recent results showing that women with online compulsive buying‐shopping disorder have higher materialistic values as compared with women reporting problematic social networking sites use (Wegmann et al. 2023).

Interestingly we found that boredom proneness and impulsivity are also associated with Negative Metacognitions about Uncontrollability and Cognitive Harm of online shopping, which in turn are associated with COS. When it comes to impulsiveness and boredom proneness, the activation of beliefs about not being able to control engagement in online shopping (i.e., negative metacognitions) may lead individuals to be entangled in shopping online as a strategy for controlling the negative affective states emanating from the perception of uncontrollability of the shopping behavior itself. These results are consistent with the findings of previous studies in which Negative Metacognitions About Uncontrollability And Cognitive Harm were found to significantly contribute to addictive behaviors, including those related to problematic online behaviors such as problematic Internet use (Spada et al. 2008), problematic online gaming (e.g., Spada and Caselli 2017), and problematic social media use (e.g., Akbari et al. 2023). This evidence may indicate that metacognitions are a transdiagnostic variable that might be helpful in explaining different addictive behaviors.

The present study has some limitations. First, the cross‐sectional nature of the study design prevents drawing causal inferences. Therefore future studies should adopt a longitudinal design to establish the direction of the detected relationships. Second, the data obtained by self‐report questionnaires might suffer from social desirability and self‐report biases. Moreover, the convenience sampling technique and a sample mainly composed of women and adult participants limits the generalizability of the results to the entire population. Thus, the present findings should be verified among more representative samples and, most importantly, among clinical samples of compulsive online shoppers. Indeed, the current sample was mainly composed of participants who declared they shop online recreationally, and therefore, testing the proposed model on individuals seeking treatment for online shopping‐related problems could be an interesting challenge for future research. Furthermore, our examination is limited to the addiction model of problematic online shopping, where the debate is still open about whether it is best construed as impulsive, compulsive, or addictive behavior.

Despite the above limitations, the current study represents the first investigation of the contribution of positive and negative metacognitions in COS and produces novel findings to understand the phenomenon. Moreover, potential clinical implications could be drawn. First, since metacognitions about online shopping have emerged as a potential key variable involved in COS, their investigation could be valuable in clinical assessment. Second, developing interventions to modify specific metacognitions associated with COS may be relevant in keeping with previous evidence showing that metacognitive interventions demonstrate their efficacy for different addictive behaviors (e.g., Caselli et al. 2016). Additionally, interventions targeted at reducing boredom proneness, impulsivity, materialism, and negative affect may be important to decrease the levels of COS (e.g., Muller, Joshi, and Thomas 2022).

In conclusion, the present findings add to the argument that the metacognitive model of addictive behaviors could be applied to the understanding of COS, akin to what has been done for other addictive behaviors. Individuals with high boredom proneness, impulsivity, materialism, and negative affect might engage in COS since they hold specific beliefs about the utility of this activity in regulating unwanted thoughts and negative affect (i.e., Positive Metacognitions about Emotional and Cognitive Regulation). Additionally, beliefs about the uncontrollability of thoughts related to online shopping and the perception of the lack of executive control over engagement in online shopping (i.e., Negative Metacognitions about Uncontrollability and Cognitive Harm) may promote the perpetuation of COS.

## Ethics Statement

The study procedures were carried out in accordance with the Declaration of Helsinki. The Institutional Review Board of the University of [first author] approved the study.

## Conflicts of Interest

The authors declare no conflicts of interest.

## Data Availability

The data that support the findings of this study are available from the corresponding author upon reasonable request.
